# Genotypic and Phenotypic Characterization of Incompatibility Group FIB Positive *Salmonella enterica* Serovar Typhimurium Isolates from Food Animal Sources

**DOI:** 10.3390/genes11111307

**Published:** 2020-11-04

**Authors:** Nesreen H. Aljahdali, Bijay K. Khajanchi, Kennedi Weston, Joanna Deck, Justin Cox, Ruby Singh, Jeffrey Gilbert, Yasser M. Sanad, Jing Han, Rajesh Nayak, Steven L. Foley

**Affiliations:** 1 Division of Microbiology, U.S. Food and Drug Administration, National Center for Toxicological Research, Jefferson, AR 72079, USA; nesreen.aljahdali@fda.hhs.gov (N.H.A.); bijay.khajanchi@fda.hhs.gov (B.K.K.); kennedi.weston@fda.gov (K.W.); joanna8deck@icloud.com (J.D.); justincox025@gmail.com (J.C.); sanady@uapb.edu (Y.M.S.); Jing.han1@fda.hhs.gov (J.H.); Rajesh.Nayak@fda.hhs.gov (R.N.); 2 Biological Science Department, College of Science, King Abdul-Aziz University, Jeddah 21551, Saudi Arabia; 3 Department of Agriculture, University of Arkansas at Pine Bluff, Pine Bluff, AR 71601, USA; 4 Office of New Animal Drug Evaluation, U.S. Food and Drug Administration, Center for Veterinary Medicine, Rockville, MD 20855, USA; ruby.singh@fda.hhs.gov (R.S.); jeff.gilbert@fda.hhs.gov (J.G.); 5 Department of Parasitology and Animal Diseases, Veterinary Research Division, National Research Centre, Giza 12622, Egypt

**Keywords:** IncFIB plasmids, *S*. Typhimurium, SNP tree, conjugation, persistence, Caco-2 intestinal epithelial cells

## Abstract

*Salmonella enterica* is one of the most common bacterial foodborne pathogens in the United States, causing illnesses that range from self-limiting gastroenteritis to more severe, life threatening invasive disease. Many *Salmonella* strains contain plasmids that carry virulence, antimicrobial resistance, and/or transfer genes which allow them to adapt to diverse environments, and these can include incompatibility group (Inc) FIB plasmids. This study was undertaken to evaluate the genomic and phenotypic characteristics of IncFIB-positive *Salmonella enterica* serovar Typhimurium isolates from food animal sources, to identify their plasmid content, assess antimicrobial resistance and virulence properties, and compare their genotypic isolates with more recently isolated *S.* Typhimurium isolates from food animal sources. Methods: We identified 71 *S.* Typhimurium isolates that carried IncFIB plasmids. These isolates were subjected to whole genome sequencing and evaluated for bacteriocin production, antimicrobial susceptibility, the ability to transfer resistance plasmids, and a subset was evaluated for their ability to invade and persist in intestinal human epithelial cells. Results: Approximately 30% of isolates (*n* = 21) displayed bacteriocin inhibition of *Escherichia coli* strain J53. Bioinformatic analyses using PlasmidFinder software confirmed that all isolates contained IncFIB plasmids along with multiple other plasmid replicon types. Comparative analyses showed that all strains carried multiple antimicrobial resistance genes and virulence factors including iron acquisition genes, such as *iucABCD* (75%), *iutA* (94%), *sitABCD* (76%) and *sitAB* (100%). In 17 cases (71%), IncFIB plasmids, along with other plasmid replicon types, were able to conjugally transfer antimicrobial resistance and virulence genes to the susceptible recipient strain. For ten strains, persistence cell counts (27%) were noted to be significantly higher than invasion bacterial cell counts. When the genome sequences of the study isolates collected from 1998–2003 were compared to those published from subsequent years (2005–2018), overlapping genotypes were found, indicating the perseverance of IncFIB positive strains in food animal populations. This study confirms that IncFIB plasmids can play a potential role in disseminating antimicrobial resistance and virulence genes amongst bacteria from several food animal species.

## 1. Introduction

As one of the major causes of foodborne illnesses throughout the world, *Salmonella enterica* is estimated to cause approximately 1.35 million infections that result in 26,500 hospitalizations and 420 deaths per year in the United States alone [1,2]. The economic cost associated with these infections and death due to salmonellosis has been estimated to be up to 3.7 billion dollars each year due to loss of work, medical care, and quality of life lost [3,4]. Ninety-five percent of *S. enterica* infections are associated with consumption of contaminated food, and most often cause gastroenteritis that is characterized by stomach cramps, diarrhea, fever, and local infections [5]. *S. enterica* includes more than 2600 serovars, and the most predominant serovar causing human disease is *S. enterica* serovar Typhimurium, according to the Centers for Diseases Control and Prevention (CDC) [6,7]. *S*. Typhimurium is a potentially invasive bacterium causing illnesses ranging from mild gastroenteritis to enteric fever [8]. During the early stages of infection, *S*. Typhimurium survives the acid barrier of the stomach and invades the intestinal epithelium, facilitated by a *Salmonella* pathogenicity island (SPI) encoded type III secretion system (T3SS) [9,10]. *S.* Typhimurium, which contributes to significant morbidity and mortality worldwide, can carry plasmids that have been shown to harbor antimicrobial resistance and virulence genes [11,12]. 

Plasmids are often present in bacterial foodborne pathogens and typically provide traits to their host including antimicrobial resistance, virulence, and metabolism of rare substances [13,14]. Plasmids are generally not necessary for cell survival under normal conditions, but they allow pathogens to evolve and adapt to new environments [15]. Many antimicrobial resistance genes are encoded on transferable plasmids, which can facilitate the horizontal transfer of resistance genes via conjugation and provide dissemination of multidrug resistant (MDR) phenotypes among different bacteria [13,14]. The emergence of MDR bacteria are a serious threat to animal health, food safety, and human health [16]. Plasmids are often classified according to their incompatibility (Inc) groups, which is a phenomenon that inhibits coexistence of plasmids with the same replication and division mechanisms in the same cell [17]. Several MDR plasmids with different Inc types have been identified in *Salmonella* [14,18]. Among them, one of the key groups of antimicrobial resistance plasmids is the IncFIB plasmids that often carry multiple resistance determinants and virulence factors that may help bacterial pathogens improve survival capability in food animal environments and may cause infections in humans [12,19]. 

Among the virulence factors present on many IncFIB plasmids, iron acquisition transport systems allow bacteria to survive in environments where iron is in limited supply [12]. *Salmonella* possess arrays of iron acquisition systems encoded on the chromosome and on plasmids [8,12,20]. These iron acquisition systems encoded on IncFIB plasmids are essential for the infection process of host cells [8,12,21]. A previous study showed that 15% of *S*. *enterica* isolates carried IncFIB plasmids that could increase *Salmonella*’s ability to colonize the chicken cecum and cause gastrointestinal disease [22]. Moreover, IncFIB plasmids carry host addiction genes, such as *ccdA*, *relB*, and *vagC*. These genes play a significant role in the stability of plasmids in the isolates [23,24]. However, much remains to be understood regarding genetic analysis of IncFIB plasmids associated with the impact on bacterial phenotype. With the advent and wide application of whole genome sequencing (WGS) over the last decade, there have been many advances in the understanding of microbial genetics. To better understand the natural history of IncFIB-positive *S.* Typhimurium isolates, this study included isolates from 1999 to 2003 and compared the findings to more recently sequenced strains. This study further characterized antimicrobial resistance and virulence in a set of IncFIB positive *S.* Typhimurium isolates, assessed the genomic and phenotypic characterization of IncFIB plasmids, and compared the relative virulence capacity of isolates and transconjugants. 

## 2. Materials and Methods

### 2.1. Bacterial Strains

Seventy-one *S.* Typhimurium isolates were identified and phenotypically characterized by standard microbiological methods [13]. These isolates were retested by PCR to confirm the presence of the IncFIB replicon [25]. Isolates originated from chicken (*n* = 33, 46%), turkey (*n* = 22, 30%), cattle (*n* = 10, 14%), swine (*n* = 4, 5.4%), and poultry water (*n* = 2, 3%) within the United States from 1999 to 2003. In addition, *Escherichia coli* J53 was used as a recipient strain for conjugation experiments [26]. Isolates were frozen at −80 °C in brain heart Infusion broth (Remel, Lenexa, KS, USA) with 20% glycerol for long-term storage. To improve our understanding of the persistence and evolution of common antimicrobial resistance genotypes over the years, we obtained eight genome sequences of recently sequenced *S*. Typhimurium isolates from GenBank. 

### 2.2. Phenotypic Testing 

#### 2.2.1. Antimicrobial Drug Susceptibility Testing (AST)

All isolates were examined for antimicrobial susceptibility testing (AST) using disc diffusion or broth microdilution following the standard Clinical and Laboratory Standards Institute (CLSI) guidelines [27,28,29,30]. Strains were tested for amoxicillin-clavulanic acid (AMC), amikacin (AMI), ampicillin (AMP), chloramphenicol (CHL), ciprofloxacin (CIP), gentamicin (GEN), kanamycin (KAN), streptomycin (STR), sulfisoxazole (SUL), trimethoprim-sulfamethoxazole (SXT), tetracycline (TET), and ceftiofur (TIO). *E. coli* ATCC 25922 was used as a quality control strain for the AST testing.

#### 2.2.2. Colicin Inhibition Assay

The ability of *Salmonella* strains to produce colicin was evaluated by assessing the ability to potentially inhibit the growth of *E. coli* J53 strain [26]. The *E. coli* J53 test strain and each of the *Salmonella* test isolates were cross streaked on tryptic soy agar with 5% sheep’s blood (blood agar, Remel). Plates were incubated at 37 °C for 16 to 18 h, after which, plates were examined for growth inhibition of *E. coli* J53 at the intersection of *Salmonella* growth. If growth was inhibited, the strain was considered positive for colicin production. 

#### 2.2.3. In Vitro Invasion and Persistence Assay

Human intestinal epithelial cells (Caco-2) were grown in Dulbecco’s Modified Eagle Medium (DMEM) containing 2.5 mM L-glutamine supplemented with 5% fetal bovine serum (FBS) and penicillin-streptomycin-amphotericin (Gibco, Gaithersburg, MD, USA), in an atmosphere of 5% CO_2_ at 37 °C. Cells were grown and maintained in the culture flask until reaching a complete monolayer. Upon confluence, cells were detached from the flask by incubating with 0.25% Trypsin-EDTA solution at 37 °C for 2–3 min and washed with DMEM and re-suspended in complete DMEM media, without antibiotics. Epithelial cells were plated in 24-well culture plates at a concentration of 5 × 10^4^ cells per well. Culture plates were incubated at 37 °C with 5% CO_2_ and 95% humidity for 24 h. Cells in one of the wells were counted using Cellometer Auto T4 (Nexcelom Bioscience, Lawrence, MA, USA). A subset of 37 *Salmonella* strains with varying PCR genotypes were selected for invasion and persistence assays.

Bacterial invasion assays were performed with a minor modification from a previously published protocol [14,31]. Caco-2 cells were infected with each of the strains at a multiplicity of infection (MOI) of 1:10. Each isolate was suspended in DMEM (~5 × 10^5^ cells/well) and added to three wells containing Caco-2 cells. Plates were centrifuged for 500 RPM for 5 min and allowed to incubate for 1 h at 37 °C in 5% CO_2_. After a 1 h incubation, cells were washed with pre-warmed PBS (pH 7.4) three times to remove extracellular bacteria and incubated with 200 µg/mL of gentamicin (Life Technology, Grand Island, NY, USA) for 1 h at 37 °C to kill the extracellular bacteria. After 1 h the cells were washed with PBS and incubated with chilled 1% Triton X-100 for 5 min at 37 °C to lyse the Caco-2 cells. Aliquots were collected, serially diluted in PBS buffer, plated onto LB agar plates and incubated at 37 °C for 18 h. The colony forming units (CFUs) were determine by plate counting. These assays were performed in three independent replicates and repeated twice for a total of nine data points. Caco-2 cells that were not infected with bacteria served as controls. 

Persistence assay: For bacterial persistence determination, cells were infected with each of the *Salmonella* isolates as described for the invasion assay. After 1 h cells were washed with pre-warmed PBS and incubated with 100 µg/mL gentamicin for 48 h at 37 °C. After 48 h cells were washed with PBS and lysed with 1% Triton X-100. The numbers of surviving bacteria were determined by plating serial dilutions of cell lysates as described above. 

#### 2.2.4. Conjugation Assay

**Figure 1:**
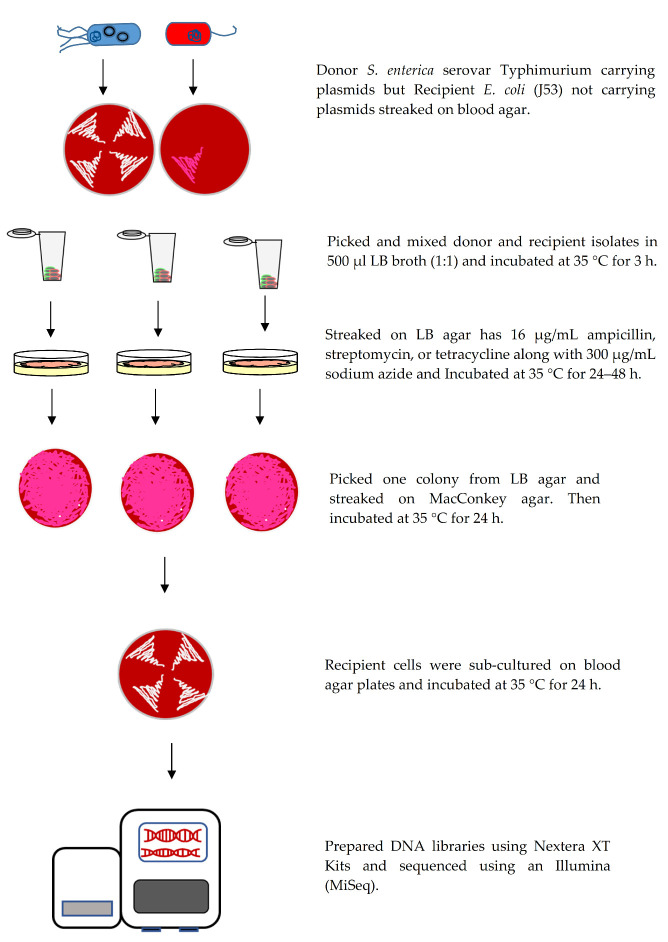
Schematic of conjugation process of Donor *S*. Typhimurium and *E. coli* J53.

*Salmonella* isolates served as potential donor cells and *E. coli* J53 strain as the recipient cell for the conjugation experiments using methods previously described by Welch et al. (2007) with minor modifications [32]. Briefly, cultures of donor and recipient cells were sub-cultured on blood agar plates and incubated at 37 °C for 24 h. The following day, donor and recipient cells were mixed in 500 μL LB broth (1:1) and incubated at 37 °C for 3 h. Transconjugants were streaked on LB agar plates containing 16 μg/mL ampicillin, streptomycin, or tetracycline along with 300 μg/mL sodium azide and incubated for up to 48 h. One colony from each plate was picked and plated on MacConkey agar (Remel) and incubated at 37 °C for 24 h. Transconjugants from MacConkey agar were sub-cultured on blood agar plates and incubated at 37 °C for 24 h. To identify which plasmids were transferred into the recipient cells, whole genome sequencing (WGS) was completed on transconjugant cells using the Illumina MiSeq as described below (Figure 1). 

### 2.3. Genotypic Testing

#### 2.3.1. Whole Genome Sequencing (WGS)

Genomic DNA of donor and transconjugant cells were extracted using a DNeasy Blood and Tissue kit (Qiagen, Valencia, CA, USA). DNA quality and quantity were measured using a Nanodrop (ThermoFisher Scientific, Grand Island, NY, USA) and Qubit BR assay (ThermoFisher Scientific). DNA libraries were constructed using 1 ng DNA from each sample using Nextera XT DNA library preparation kits (Illumina, San Diego, CA, USA). Samples were multiplexed using combinations of two indexes of Nextera XT Index Kit (Illumina). DNA samples were diluted, denatured, and loaded on an Illumina MiSeq instrument with a 2 × 250 pair-end format. Samples were sequenced in multiple batches.

#### 2.3.2. Single Nucleotide Polymorphism (SNP) Analysis 

**Figure 2:**
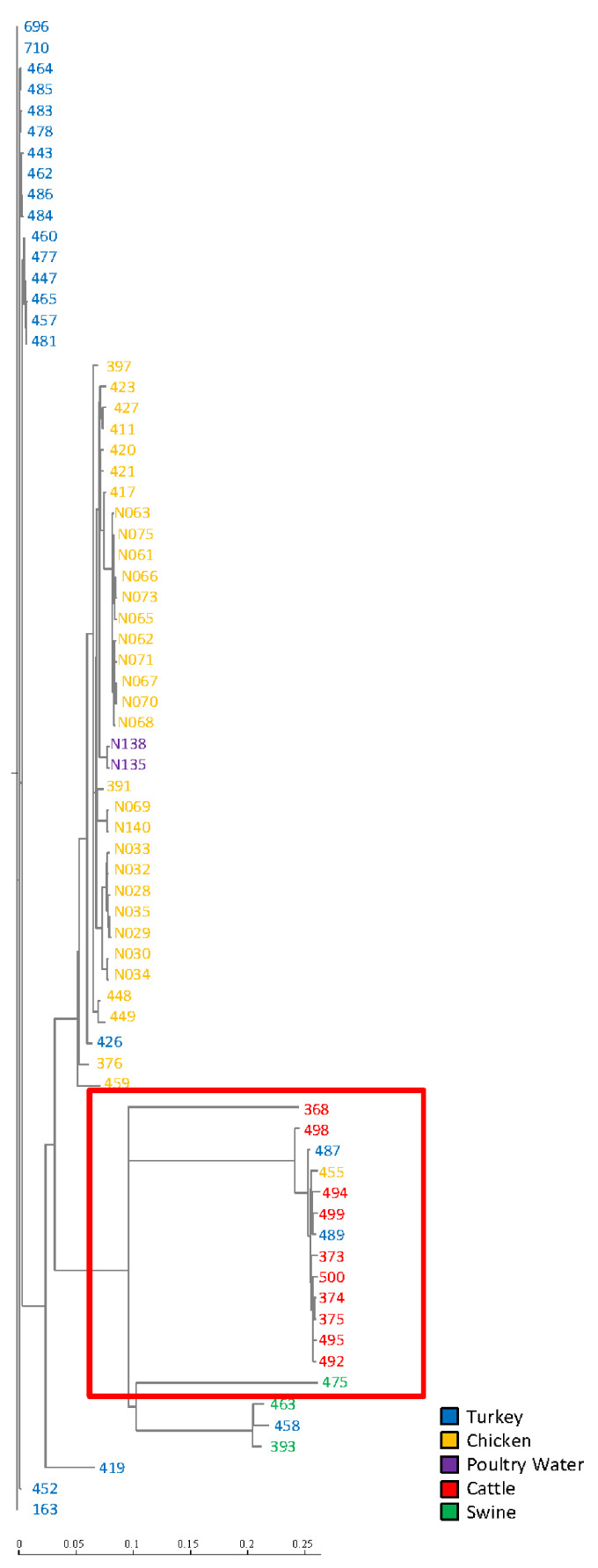
Phylogenetic analysis for 71 S. Typhimurium. SNP analysis indicates most of the isolates clustered together based on the host origin and some iosolates grouped together based on geographic region. The red box indicates *spv* genes were found in these isolates. The numbers on the scale present the percentage of genetic variation.

Phylogenetic analysis of the sequenced *Salmonella* genomes (*n* = 71) was carried out to identify the core genome of isolates and build a phylogenetic tree using single nucleotide polymerase (SNP). A second tree was built incorporating sequence data from eight IncFIB-positive *S.* Typhimurium isolates collected from 2005–2018 to determine the relatedness of the more recently collected isolates to those collected earlier. These eight presumptive IncFIB-positive *S.* Typhimurium isolates were identified by BLAST searching with the IncFIB replicon sequence against *S.* Typhimurium genomes. Whole-genome sequence (WGS) data from isolates which originated from food animal sources within the United States after 2003, were downloaded from NCBI for subsequent SNP analyses. WGS-based single nucleotide polymorphism (SNP) analyses was performed using the FDA Center for Food Safety and Applied Nutrition (CFSAN) SNP pipeline as described by Davis et al. [33] installed on GalaxyTrakr (https://www.galaxytrakr.org) [34]. Briefly, a matrix of SNPs from WGS FastQ data were created using reference-based alignments by CFSAN SNP pipeline. Phylogenetic trees were constructed using the best scoring maximum likelihood (ML) SNP tree with randomized axelerated ML (RAxML) using a GTRGAMMA model with default parameter settings on GalaxyTrakr [35]. The SNP tree was generated by uploading FASTQ data of forward and reverse files of 71 isolates into GalaxyTrakr from a local computer (Figure 2). SNP trees were generated after downloading FASTQ data from 79 isolates from SRA submission using the SRA toolkit on GalaxyTrakr (Supplemental Figure S1). In both trees, SNPs matrix was built using *Salmonella enterica* serovar Typhimurium str. LT2 as reference genome (accession: NC_003197.2).

#### 2.3.3. Bioinformatic Analyses

In this study we used a variety of sequence analysis applications to identify antimicrobial resistance and virulence genes encoded on plasmids. Genome sequences from 71 *S.* Typhimurium isolates (donors) and 24 transconjugants were trimmed, and de novo assembly was completed using CLC Genomics Workbench (version. 9.0, Qiagen, Redwood City, CA, USA). FASTA files of sequence assemblies from each strain were analyzed using PlasmidFinder (version 2.1) and ResFinder (version 4.1) to identify predicted plasmids and antimicrobial resistance genes, respectively [36,37]. Pathosystems Resource Integration Center (PATRIC-database) [38] and an in-house *Salmonella* virulence factor database curated from PATRIC, Victor, VFDB, and the literature (NCTR Virulence Factor Database) were queried to identify putative virulence genes. Virulence genes were extracted from the in-house database (NCTR-database), transformed to binary data and imported into BioNumerics for phylogenetic analysis using Dice Coefficients and UPGMA (Applied Maths, Austin, TX, USA). The Basic Local Alignment Search Tools (BLAST) were used to identify plasmid transfer-associated genes and colicin genes.

### 2.4. Statistical Analysis

Data were imported into Excel (Microsoft, Redmond, WA, USA) and two-tailed T-tests were used to determine statistical differences between cell survival of invasion and persistence ratio, with a *p*-value ≤ 0.05 considered as a significant difference between two groups. 

## 3. Results

**Figure 3:**
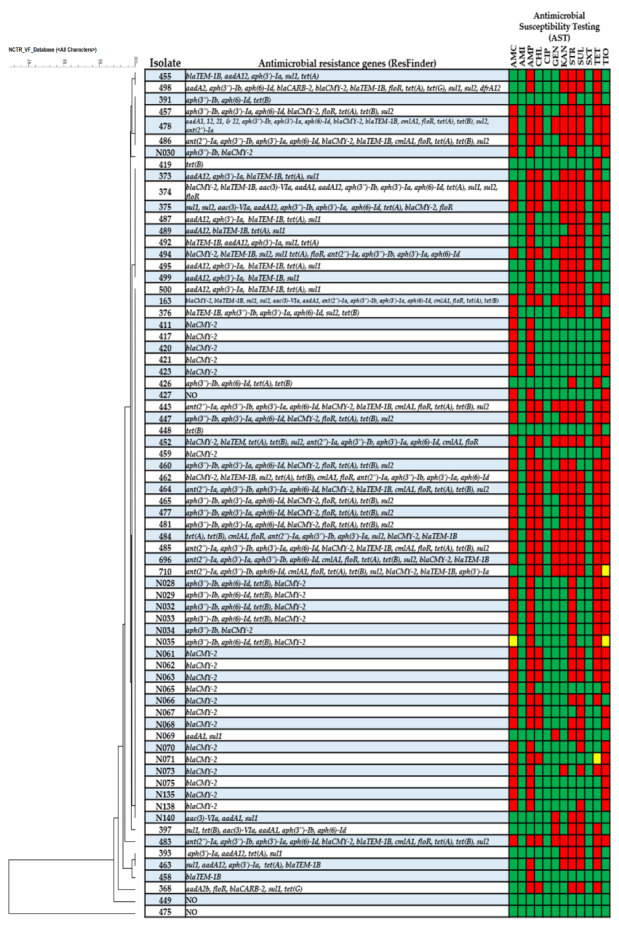
Virulence gene profile comparison and antimicrobial resistance characterization of the 71 *Salmonella* isolates. The dendrogram on the left is based on the phylogenetic comparison of 491 virulence or putative virulence genes described in Table S3. The columns to the right display the resistance genes identified and antimicrobial susceptibility testing results, where a red box indicates resistance, a green box susceptible and a yellow box indicates intermediate susceptible.

The study used multiple experiments to analyze genotypic and phenotypic characteristics associated with IncFIB plasmid-positive *Salmonella enterica* serovar Typhimurium isolates originating from different food animal sources. Sequencing assemblies of all isolates were submitted to NCBI under the accession numbers listed in Table 1. Using SNP-based phylogenetic analyses, the genetic structure of most *S*. Typhimurium isolates clustered together based on host origin. Isolates that originated from chickens typically clustered to those isolates collected from turkey sources. These poultry-associated isolates were in distinctly different phylogenetic clades from those isolates collected from swine and cattle. Many of these isolates originating from chickens and poultry water were collected from West Virginia and clustered together, which likly indicated that same or similar strains circulating in the close geographic region. Likewise, isolates 373, 374, and 375 originating from cattle in Michigan grouped together; however, these also clustered with other strains from cattle and poultry indicating that this genotype may have been more widely distributed (Figure 2). Furthermore, the SNP based evolutionary tree showed *S*. Typhimurium isolates of the current study were phylogenetic clades from eight genome sequences of the more recently sequenced *S*. Typhimurium isolates (Figure S1**).** These isolates shared a high degree of genetic relatedness and carried similar virulence and antimicrobial resistance genes, including *aph(3″)-Ib, aph(6)-Id, bla_TEM-1B_, bla_CMY-2_, sul2, tet(A), tet(B), tet(G), sul1,* and *sul2* (Figure 3; Tables S1 and S2).

**Figure 4:**
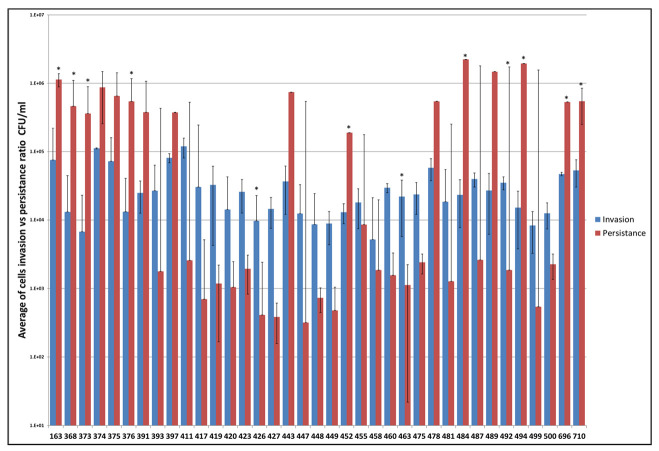
Results of the invasion and persistence studies of 37 *S*. Typhimurium. The bacterial invasion (blue bars) indicated the means of ratio cells detected after a 1 h invasion and the bacterial persistence (red bars) indicted the means of the ratio cells detected after a 48 h persistence. The error bars show the standard error of means. (*) determined the significant differences between two groups.

All 71 strains were used as donors for conjugation and 24 strains (34%) were able to generate transconjugants. Each isolate and transconjugant underwent WGS analyses to identify antimicrobial resistance genes, putative virulence factors, bacteriocin genes and transfer-associated genes. Results of phylogenetic analyses with BioNumerics based on the presence of the virulence factors for 71 *S*. Typhimurium are displayed in Figure 3 and Table S3. The NCTR virulence factor database identified putative virulence factors and the heatmap indicated the presence or absence of specific virulence genes for 71 isolates. Most of the isolates had very similar virulence factor profiles, with the noted exception of isolate 475, which was most distant from the other isolates. Of note 475 carried operons for multiple fimbriae (*pef* and *sef*) and sugar efflux transport (*set*) that were not present in any of the other isolates and carried the *spv*-containing virulence plasmid that was present in 14 (20%) of the isolates (Table S3). The invasion and persistence results for 37 isolates that can invade Caco-2 cells are presented in Figure 4. Nine of 37 (24%) isolates displayed significantly higher cell numbers at 48 h of persistence, compared to the 1 h invasion period. Likewise, seven isolates (19%) displayed increased bacterial cell numbers after 48 h of infection which did not reach significant differences between two groups. Conversely, three of 37 (8%) isolates demonstrated significantly higher bacterial cell counts after 1 h, compared to a 48 h incubation period, while 18 out of 37 (49%) isolates exhibited higher numbers of bacterial cells after 1 hr of invasion which did not reach statistical significance. When looking at the invasion and persistence results compared to either the virulence factor profiles and SNP analyses, there is not a clear trend that explains the variability in the persistence findings. All of the isolates contained the genes encoding *Salmonella* pathogenicity island-1 (SP-1) encoded type III secretion system (T3SS), which facilitated their ability to invade the host cells and *Salmonella* pathogenicity island-2 (SP-2) associated with the persistence of the *Salmonella* in host cells (Tables S1 and S3). Further efforts to understand the variability in persistence are needed, for example is there a role of the co-localization with other plasmids in persistence. Interestingly of the 13 strains tested that carried both IncFIB and IncA/C plasmids, six had significantly greater persistence and four displayed increased bacterial cell numbers after 48 hr of infection which did not reach significance. These numbers are 67% (6/9) and 57% (4/7) of the strains that fell into these persistence categories, respectively. Two of the remaining three IncA/C positive strains had fairly large standard error bars with the persistence range larger than that of the invasion. Whether there is a link between the plasmids and persistence merits further evaluation. 

The results of colicin inhibition and detection of the colicin-associated genes for all isolates are shown in Table 2. Twenty-one isolates (29%) were able to inhibit the growth of *E. coli* J53. All isolates that inhibited *E. coli* J53 growth carried the channel-forming colicin genes *cia* and *cib* and the colicin b immunity (*imm)* genes, while 17 (23%) were positive for the *cva* colicin production gene, with isolates N065, N068, N071, and N073 lacking *cva*. Among the conjugation-related genes, the WGS data using PATRIC and BLAST identified *pil* and *tra* genes, which are the most representative genes in the isolates. Most of the isolates contained *traT* (*n* = 68, 95%), *traJ* (*n* = 38, 53%), and (*n* = 32, 45%) carried *pilPM*, followed by *pilJ* (*n* = 31, 44%), and *pilI* (*n* = 5, 7%). Only 24 (34%) strains were able to generate transconjugants using the methods employed in this project (Table 2). 

WGS analyses using PlasmidFinder software confirmed that all isolates contained IncFIB plasmids along with multiple other plasmid replicon types, including IncFIC(FII) (*n* = 34, 48%), IncFIA (*n* = 23, 23%), IncFII(pCoo) (*n* = 19, 27%), IncFII(S) (*n* = 14, 20%), ColpVC (*n* = 44, 62%), IncI1 (*n* = 32, 45%), IncA/C2 (*n* = 20, 28%), IncX1 (*n* = 11, 15%), IncHI2 (*n* = 8, 11%), IncHI2A (*n* = 8, 11%), and IncX4 (*n* = 3, 4.2%). PlasmidFinder analyses confirmed that seventeen transconjugants contained IncFIB plasmids (*n* = 17, 71%) along with other plasmid replicon types, including IncFIA (*n* = 16, 67% ), IncFII(pCoo) (*n* = 16, 67%), IncI1 (*n* = 5, 21%), IncA/C2 (*n* = 3, 12.5%), IncColpVC (*n* = 2, 8.3%), and IncX4 (*n* = 1, 4%). These results demonstrate that the distribution of plasmids to the recipient *E. coli* J53 can be variable. In some cases the plasmids, like the IncFIB plasmids, are not being transferred conjugally even though the donor carried the plasmid type (Table 3).

PATRIC and BLAST software identified putative virulence factors, including iron acquisition genes. All the *Salmonella* strains were positive for Salmochelin siderophore system genes (*iro*), but none of the transconjugants carried them. *Salmonella* isolates carried the aerobactin iron acquisition system *iucABCD* (*n* = 53 75%) and *iutA* (*n* = 67, 94%) genes, while some transconjugants also contained *iucABCD* (*n* = 13, 54%) and *iutA* (*n* = 16, 66%). The regulated iron transporter system (*sitABCD*) was found in 54 isolates (76%), followed by *sitAB* (*n* = 17, 24%), and 13 transconjugants carried *sitABCD* (54%). Remarkably, seven of transconjugants lacked the iron acquisition genes because the IncFIB plasmids associated with iron acquisition genes were not transferred to the transconjugants generated (Table 3). In addition, virulence factor analyses identified the *Salmonella* plasmid virulence gene (*spv*) in some isolates (*n* = 14, 20%), but none of transconjugants (Table 4 andTable S3). Most *spv* was found in cattle (*n* = 10), and other sources, including turkey (*n* = 2), swine (*n* = 1), and chicken (*n* = 1); each of these isolates, with the exception of 475, clustered together and shared a common ancestor (LCA), as shown in the red box Figure 2.

In addition, WGS data was analyzed using ResFinder software to identify antimicrobial resistance genes commonly associated with IncFIB plasmid positive *S*. Typhimurium isolates. ResFinder software revealed that 24 of the transconjugants carried multiple antimicrobial resistance genes that were obtained from the donor cells via conjugation, listed in Table 4. AST identified *Salmonella* isolates that were resistant to AMP (*n* = 59, 83%), AMC (*n* = 46, 65%), TIO (*n* = 43, 61%), CHL (*n* = 22, 31%), GEN (*n* = 15, 21%), KAN (*n* = 31, 44%), STR (*n* = 46, 65%), SUL (*n* = 36, 51%), and TET (*n* = 43, 61%). Only two strains showed intermediate susceptibility to TIO (*n* = 2, 4%) followed by TET (*n* = 1, 2%), and AMC (*n* = 1, 2%). For the corresponding resistance genotypes(*n* = 48, 68%) strains carried *bla_CMY-2_*, followed by *bla_TEM-1B_* (*n* = 22, 31% ), *bla_CARB-2_* (*n* = 2, 3%), *bla_TEM_* (*n* = 1, 2%), *floR* (*n* = 23, 32%), *cmlA1* (*n* = 12, 17%), *ant(2″)-Ia* (*n* = 12, 17%), *aac(3)-Via* (*n* = 5, 7%), *aph(3″)-Ia* (*n* = 31, 44%), *aph(3″)-Ib* (*n* = 32, 45%), *aph(6)-Id* (*n* = 30, 42%), *aadA12* (*n* = 9, 16%), *aadA1* (*n* = 4, 7.3%), *aadA2b* (*n* = 1, 2%), *aadA21* (*n* = 1, 2%), *aadA22* (*n* = 1, 2%), *sul1* (*n* = 18, 25%), *sul2* (*n*= 21, 30%), *tet(A)* (*n* = 32, 45%), *tet(B)* (*n* = 28, 39%), and *tet(G)* (*n* = 2, 3%), presented in Figure 3. 

## 4. Discussion

*S*. *enterica* have adopted diverse mechanisms in order to survive and proliferate within eukaryotic cells. *Salmonella* primarily infect the host through the consumption of contaminated animal-derived foods [1,39]. *S. enterica* harbors plasmids that play vital roles in the dissemination of antimicrobial resistance and virulence genes among bacteria [11]. In this study all of the *S*. Typhimurium strains carried IncFIB plasmids, one of the key features of many IncFIB plasmids is that they often possess iron acquisition genes (e.g., sit and aerobactin operons), which can play a significant role in persistence of *Salmonella* in the host cell, where iron is in limited supply [12]. These genes can facilitate the chelation of iron from the host during the successful infection. Additionally, IncFIB plasmids can contain antimicrobial resistance and virulence factors that have been associated with pathogenic *S*. Typhimurium [11,12,13,14,19]., which likely contribute to increase virulence of *S*. Typhimurium during infection of host cells due to carrying iron acquisition genes. 

To understand the evolutionary relatedness of the IncFIB-positive *S*. Typhimurium strains in the current study, SNP analysis revealed the genetic structure of *S*. Typhimurium isolates clustered in three monophyletic groups based on host origin, meaning that all descendants of each host origin share the LCA, with the exception of a few isolates. Similarly, in the previous report, SNP analysis of genomes of *S*. *enterica* strains from serotypes Heidelberg, Typhimurium, and Kentucky clustered together based on the specific serotype [12]. To understand the evolutionary and temporal context of the IncFIB plasmid positive isolates in this study, it was necessary to assess the study results in the context of more recently sequenced *S*. Typhimurium isolates. We obtained eight genome sequences of more recently sequenced *S*. Typhimurium isolates from a public database. The FASTQ files were downloaded using the SRA tool kit on GalaxyTrakr and built the SNP tree incorporated with the isolates from the current study using the CFSAN SNP pipeline. The SNP based evolutionary tree showed *S*. Typhimurium isolate 368 that originated from cattle in 1999 was grouped in a phylogenetic clade with isolates originating from swine in 2016 and cattle in 2010 (Figure S1 green box). These isolates carrying IncFIB shared a high degree of genetic relatedness and two of these isolates carried similar antimicrobial resistance genes, including *floR*, *bla _CARB-2_, aadA2b, sul1,* and *tet(G)*, with the strain isolated in 2010 lacking AMR genes. Likewise, *S*. Typhimurium isolated from turkey in 2012 formed a clade with strains 463, 458, and 393 which originated from turkey and swine in 1999 (Figure S1 red box). ResFinder revealed that these isolates 463, 458, and 393 carried identical antimicrobial resistances genes, including *bla_TEM-1B_*, *tet(A)*, and *sul1,* while a strain isolated from turkey in 2012 carried *tet(B)* and *sul2*. Also, we found that these isolates were grouped in a phylogenetic clade with those from poultry-associated strains isolated in 2005, 2011, and 2016. These strains shared the LCA, and they carried similar AMR genes, including *aph(3″)-Ib, aph(6″)-Id, sul2, tet(A)*, and *tet(B)* (Figure S1 blue box). A *S*. Typhimurium strain isolated from turkey in 2018 branched separately from the isolates originated from turkey and cattle in 1999 (Figure S1 brown box). Also, we found that the strain from swine isolated in 1999 closely clustered with *S*. Typhimurium carrying the IncFIB plasmid isolated from swine in 2018. These strains demonstrated a high degree of genetic relatedness and carried the same antimicrobial resistance gene *tet(B)* (Figure S1 purple box). Overlapping genotypes of isolates from the late 1990s with those isolated as recently as 2018 indicate that multiple IncFIB-positive *Salmonella* strains have likely persisted in livestock environments over multiple decades and can potentially lead to continued problems with antimicrobial resistance.

Several studies have indicated that IncFIB plasmids likely contribute to increased colonization in the cecum of poultry, which may help explain their persistence in food animal populations [12,40]. After colonization, *Salmonella* can compete with other bacteria by producing antimicrobials, such as bacteriocins, which are often called colicins [41]. In the inflamed gut it was found that the pCol1B9-plasmid was able to transfer from *S.* Typhimurium to commensal *E. coli* [42]. This plasmid encodes for colicin Ib (*cib*) and immunity (*imm*), which increase the fitness of *S*. Typhimurium in competition with commensal colicin-sensitive *E. coli* [42]. The structural and functional properties of colicin Ib are closely related to colicin Ia [43]. In our earlier work, IncI1 plasmid colicin-associated *cib* and *imm* were detected in different serotypes of *Salmonella*. When colicin genes were detected, the corresponding inhibition phenotype was observed in *E. coli* J53 [13]. In the current study, 21 strains (29%) were able to inhibit growth of colicin-negative *E. coli* J53. The WGS analysis using the NCBI database revealed that 21 strains were positive for *cia, cib, cvaBC*, and *imm* genes, compared to other strains.

To survive at the site of infection, *Salmonella* obtains scarce nutrients from the host environment. One essential nutrient is iron, which is known as a cofactor for various metabolic enzymes, acts as catalyst in electron transport processes, and regulates gene expression [12,44]. *S*. Typhimurium is unable to use heme as an iron source [45]. However, it possesses several iron uptake systems, such as *sitABCD*, *iroBC*, and *iucABCD-iutA* [8,12,46]. Previous studies indicated that these systems play a significant role in iron acquisition and facilitate survival under low iron conditions of pathogenic bacteria [8,12,46]. It was demonstrated that deletion of the sit system, which encoded on the chromosome, decreased the ability of *S*. Typhimurium to cause infection in the animal host [8]. The study showed that *sit* and aerobactin systems located on an IncFIB plasmid resulted in an increased the ability of *S. enterica serotypes* to colonize chickens [22] and an increased persistence of *S*. Typhimurium in human intestinal epithelial cells (Caco-2) [12]. A recent study found that *sitA* encoded on the chromosome and IncFIB plasmids were overexpressed in *S*. Typhimurium, compared to the transconjugants [20]. It was found that *sitABCD* and *iroBC* genes are located on the chromosome and the IncFIB plasmid [8,12], but *iucABCD-iutA* is usually only plasmid encoded [11,12]. In the present study we found that isolates possessed *sitAB*, *iroNB*, and *iucABCD-iutA* genes. Thus, the IncFIB plasmid encoded iron acquisition systems associated with biological functions, and chromosome-encoded iron acquisition systems play a significant role in chelating iron from the host to establish successful infection as previously mentioned [8,12,20] (Table S1). Iron acquisition systems may be potential targets for anti-infective approaches to control the colonization and persistence with *Salmonella* and other pathogens [47].

*S.* Typhimurium harbors *Salmonella* pathogenicity island-1 (SPI-1) encoded type III secretion system (T3SS) and *Salmonella* pathogenicity island -2 (SPI-2) encoded T3SS, which facilitate attachment, invasion, and internalization of the cells [48]. In the present study, WGS using NCTR and PATRIC databases confirmed that all isolates contained *S*PI-1 and *S*PI-2 encoded on T3SS (Table S1). *Salmonella* mainly depends on gene products encoded on *S*PI-1 to invade cells. Among them are *Salmonella* invasive proteins (Sips) and *Salmonella* outer proteins (Sops), which play a significant role in altering the actin cytoskeleton of the cell and promoting the *Salmonella*-containing vacuole (SCV) [49]. Within the SCV, *Salmonella* expresses genes encoded on *S*PI-2 whose proteins are important for survival and proliferation in epithelial cells [50]. Moreover, it was reported that *S*. Typhimurium is known to proliferate to large numbers in the cytosol of epithelial cells at later stages of infection [51]. Here, we used Caco-2 cells, which are derived from human intestinal epithelial cells [52]. We observed a significant increase of some persistent bacterial cells (*n* = 10, 27%) at 48 h, compared to invasion of the bacterial cells (*n* = 2, 5%) of *S.* Typhimurium, which is consistent with a previous study showing a significant increase in persistent cell counts after 48 h, compared to invasion cell counts after 1 h of IncI1 positive *Salmonella* of different serotypes [14]. Furthermore, it was reported that *sifA* and SPI-2 are involved in maintenance of the vacuolar membrane and intracellular replication in vivo [53]. Another study found that *Salmonella* deficient for the gene *sifA* cannot maintain vacuolar integrity and subsequently replicate in the cytosol of epithelial cells [54]. In this study, WGS analyses using the PATRIC and NCTR Virulence Factor database revealed that all isolates harbored the gene *sifA*, and *S*PIs encoded T3SS (Table S1). 

Horizontal gene transfer (HGT) can occur between *Salmonella* and members of *Enterobacteriaceae* via conjugation, in which a recipient cell receives genetic elements from a donor cell by cell-to-cell contact through conjugative pili [16]. In the present study, most isolates of *S*. Typhimurium were positive for *traJT* and *pilJPM* genes, which are representative genes associated with the conjugal transfer function. Twenty-four isolates generated transconjugants when mated with the recipient *E. coli* J53. We found that most transconjugants harbored IncFIB plasmids along with multiple other plasmid replicon types and carried multiple antimicrobial resistance genes that were transferred from the donor. Some of plasmids were not transferable to *E. coli* J53 and this could be due to plasmid size, the sources of animal foods, mutations in *pil*, *tra* or regulatory genes and/or conjugation method employed that can impact efficiency (broth versus solid mating, incubation temperature, and donor/recipient ratio). The stability of IncFIB plasmids in bacteria is due in large part to host addiction genes, including *ccdA*, *relB*, and *vagC* [23,24]. Thus, the IncFIB plasmids were able to disseminate horizontally among bacteria and were maintained in bacterial populations [12]. An earlier study demonstrated that in vivo transfer of an IncFIB plasmid containing a class 1 integron harboring gene cassettes *dfrA1-aadA1* occurred from *Salmonella* to *E. coli* [55]. *Salmonella* was able to transfer multiple resistance genes on transferable plasmids to commensal *E. coli* in an in vivo assay [56,57]. Likewise, in an in vitro test, an IncI1 plasmid carrying *tetA, bla_CMY_, aacC, aadA1*, and *sul1* was shown to be conjugatively transferred from *Salmonella* to *E. coli* J53 [13].

The WGS analysis using ResFinder revealed several antimicrobial resistance genes, which corresponded to the predicted resistance phenotype in most strains. Some of the more common resistance genes identified in the IncFIB-positive isolates included *bla_CMY-2_, bla_TEM-1B_, sul, aac(3)-VIa, ant(2″)-Ia, aadA1, aph(3″)-Ib, aph(6)-Id, aph(3′)-Ia, cmlA1, floR,* and *tet*. These genes encode resistance to AMC, AMP, TIO, SUL, GEN, STR, KAN, CHL, and TET. When the resistance phenotypes were compared to several of the genes noted, there was a positive concordance of gene presence and resistance phenotype in several strains, with few observed exceptions. However, nine strains were positive for *bla_TEM-1B_* and two were positive for *bla_CMY-2_* but were still susceptible or intermediate susceptible to AMC. Also, we found five strains (7%) that were positive for *bla_CMY-2_* genes; however, they were still susceptible or intermediately susceptible to TIO. In a small number of cases, susceptible bacteria to AMP, CHL GEN, and SUL were positive for *bla_TEM-1B_* (*n* = 1, 1.4%), *floR* (*n* = 1, 1.4%), *ant (2”)-la* (*n* = 1, 1.4%), *aac (3)-Via* (*n* = 1, 1.4%) and *sul* (*n* = 1, 1.4%), respectively. This study is consistent with a previous study that found some susceptible strains to TET and SUL were positive for *tetA* and *sul1* genes on IncI plasmid-positive *Salmonella* [13]. The difference between genotype and phenotype in these strains might be due to mutations that reduce the expression of genes. Thus, point mutations in genes can confer susceptibility to selected antimicrobial agents. Despite the discovery of many antimicrobial resistance genes and genetic transfer mechanisms, the non-genetic mechanisms mediated by small molecules can alter the antibiotic susceptibility of bacteria cells [58]. Indole, which is produced by Gram-positive and Gram-negative bacteria, can also protect the bacterial cells from antibiotic damage [59]. It was found that certain *E. coli* produce a higher level of indole in the presence of ampicillin and kanamycin [59]. Furthermore, indole can mediate upregulation of multidrug efflux pumps by inducing the expression of various multidrug genes and enhancing the efflux of antibiotics [60,61]. Efflux pumps allow bacteria to regulate their internal environment by removing toxic materials, including antimicrobial agents [62]. In the present study we found that some strains were resistant to SUL (*n* = 9, 16%), followed by CHL (*n* = 8, 15%), TET (*n* = 6, 11%), STR (*n* = 5, 9%), KAN (*n* = 2, 3%) AMC (*n* = 1, 2%), AMP (*n* = 1, 2%), TIO (*n* = 1, 2%), and GEN (*n* = 1, 2%), and yet did not have a corresponding resistance gene detected. Resistance of these strains could be due to the intrinsic resistance of antibiotic response as previously suggested [60,61,62]. In the WGS data from the eight reference strains analyzed, ResFinder revealed that these strains possessed resistance genes similar to those detected in strains from the study including *aadA12, aadA2, aph(3′)-Ia, blaTEM-1B, sul1, sul2, tet(A), tet(B), tet(G), aph(3″)-Ib, aph(6)-Id, blaCMY-2* and *floR* (Table S2). 

## 5. Conclusions

*Salmonella* is a potentially invasive pathogen that causes many human illnesses. During infection, *Salmonella* induces several virulence genes required to survive and compete with other bacteria. Many of these genes are encoded on transferable plasmids, which can facilitate the horizontal transfer of virulence genes, along with multiple antimicrobial resistance genes. The IncFIB plasmids, which often carry multiple resistance determinants and virulence factors, were detected in *S*. Typhimurium. This study was undertaken to assess the genomic and phenotypic characterization of IncFIB positive *S*. Typhimurium isolates from different animal-derived foods. Results of this study showed that IncFIB plasmids along with other plasmid replicon types can contribute to antimicrobial resistance, virulence factors, and persistence in intestinal epithelial cells. Thus, IncFIB plasmids represent a likely threat to human and animal health due to the transfer of antimicrobial resistance and virulence genes to susceptible bacteria. WGS-based sequence analyses associated with phenotype analysis of IncFIB plasmids facilitate better understanding of the genetics of antimicrobial resistance and virulence genes, which will help in the design of specific intervention strategies to stop plasmid spread among bacteria. 

## Figures and Tables

**Table 1:** Isolate information for 71 *Salmonella enterica* serovar Typhimurium.

Isolate	Source	Isolation Location	Year	GenBank Accession
163	Turkey	OH	1999	LSZD00000000
368	Cattle	GA	1999	PDNO00000000
373	Cattle	MI	1999	PDNN00000000
374	Cattle	MI	1999	VTSM00000000
375	Cattle	MI	1999	VTSL00000000
376	Chicken	GA	1999	PDNM00000000
391	Chicken	USA	1999	VTSK00000000
393	Swine	USA	1999	VSXW00000000
397	Chicken	USA	1999	LYRR00000000
411	Chicken	USA	1999	VSXV00000000
417	Chicken	USA	1999	VSXU00000000
419	Swine	USA	1999	VSXT00000000
420	Chicken	USA	1999	VSXS00000000
421	Chicken	USA	1999	VSXI00000000
423	Chicken	USA	1999	VSXR00000000
426	Turkey	USA	1999	PDNK00000000
427	Chicken	USA	1999	VSXQ00000000
443	Turkey	USA	1999	VSXP00000000
447	Turkey	USA	1999	VSXO00000000
448	Chicken	USA	1999	VSXN00000000
449	Chicken	USA	1999	VSXM00000000
452	Turkey	USA	1999	LYRS00000000
455	Chicken	USA	1999	VSXL00000000
457	Turkey	USA	1999	VSXH00000000
458	Turkey	USA	1999	VSXK00000000
459	Chicken	USA	1999	VTSH00000000
460	Turkey	USA	1999	VTSI00000000
462	Turkey	USA	1999	VTSU00000000
463	Swine	USA	1999	PHGW00000000
464	Turkey	USA	1999	VSXG00000000
465	Turkey	USA	1999	VTSG00000000
475	Swine	USA	1999	VSXJ00000000
477	Turkey	USA	1999	VTSF00000000
478	Turkey	USA	1999	LYRT00000000
481	Turkey	USA	1999	VSXF00000000
483	Turkey	USA	1999	VTSD00000000
484	Turkey	USA	1999	PDOI00000000
485	Turkey	USA	1999	VSXE00000000
486	Turkey	USA	1999	VTSC00000000
487	Turkey	USA	1999	VSXD00000000
489	Turkey	USA	1999	VTSB00000000
492	Cattle	USA	1999	PDOJ00000000
494	Cattle	USA	1999	PDOK00000000
495	Cattle	USA	1999	VTSA00000000
498	Cattle	USA	1999	VSXC00000000
499	Cattle	WV	1999	VTRZ00000000
500	Cattle	USA	1999	VSXB00000000
696	Turkey	USA	1999	LXHA00000000
710	Turkey	ND	1999	LXGZ00000000
N028	Chicken	WV	2003	VSWY00000000
N029	Chicken	WV	2003	VSXA00000000
N030	Chicken	WV	2003	VSWX00000000
N032	Chicken	WV	2003	VSWZ00000000
N033	Chicken	WV	2003	VTRY00000000
N034	Chicken	WV	2003	VSWW00000000
N035	Chicken	WV	2003	VSWV00000000
N061	Chicken	WV	2003	VSWU00000000
N062	Chicken	WV	2003	VTRX00000000
N063	Chicken	WV	2003	VSWT00000000
N065	Chicken	WV	2003	VSWS00000000
N066	Chicken	WV	2003	VSWR00000000
N067	Chicken	WV	2003	VSWQ00000000
N068	Chicken	WV	2003	VSWP00000000
N069	Chicken	WV	2003	VSWO00000000
N070	Chicken	WV	2003	VSWN00000000
N071	Chicken	WV	2003	VSWM00000000
N073	Chicken	WV	2003	VTRW00000000
N075	Chicken	WV	2003	VSWL00000000
N135	Poultry Water	WV	2003	VSWK00000000
N138	Poultry Water	WV	2003	VSWJ00000000
N140	Chicken	WV	2003	VSWI00000000

USA: State-level information not available; OH: Ohio; GA: Georgia; MI: Michigan; ND: North Dakota; WV: West Virginia.

**Table 2:** Phenotypic and genomic results of colicin and conjugation assays for 71 isolates.

Isolate	Plasmid Transfer-Associated Genes		Conjugation	Colicin Genes				Colicin Inhibition
	Type IV Pilus Biogenesis Protein	Tra Genes		Colicin Ia Synthesis Protein	Colicin Ib Protein	Colicin V Synthesis Protein	Colicin Ib Immunity Protein	
163	*pilJPM*	*traTJ*	YES	*cia*	*cib*	N	*imm*	NO
368	N	*traT*	NO	N	N	N	N	NO
373	N	*traT*	NO	N	N	N	N	NO
374	*pilIJPM*	*traTJ*	NO	N	N	N	N	NO
375	*pilIJPM*	*traTJ*	YES	N	N	N	N	NO
376	N	*traT*	NO	N	N	N	N	NO
391	N	*traT*	NO	N	N	*cvaABC*	N	NO
393	*pilIJPM*	*traTJ*	YES	N	N	N	N	NO
397	*pilJPM*	*traJ*	NO	*cia*	*cib*	N	N	NO
411	*pilJPM*	*traTJ*	NO	*cia*	*cib*	*cvaBC*	*imm*	YES
417	*pilJPM*	*traTJ*	NO	*cia*	*cib*	N	*imm*	NO
419	*N*	N	NO	N	N	N	N	NO
420	*pilJPM*	*traTJ*	NO	*cia*	*cib*	*cvaBC*	*imm*	YES
421	*pilJPM*	*traTJ*	NO	*cia*	*cib*	*cvaABC*	*imm*	YES
423	*pilJPM*	*traTJ*	NO	*cia*	*cib*	*cvaABC*	*imm*	YES
426	*pilPM*	*traTJ*	NO	N	N	*cvaABC*	N	YES
427	N	*traT*	NO	N	N	*cvaABC*	N	NO
443	N	*traT*	YES	N	N	N	N	NO
447	N	*traT*	YES	N	N	N	N	NO
448	N	*traT*	NO	N	N	N	N	NO
449	N	*traT*	NO	N	N	N	N	NO
452	N	*traT*	YES	N	N	N	N	NO
455	N	*traT*	NO	N	N	N	N	NO
457	N	*traTJ*	YES	N	N	N	N	NO
458	*pilIJPM*	*traTJ*	YES	N	N	N	N	NO
459	*pilJPM*	*traTJ*	YES	*cia*	*cib*	*cvaABC*	*imm*	YES
460	N	*traT*	YES	N	N	N	N	NO
462	N	*traT*	YES	N	N	N	N	NO
463	*pilIJPM*	*traTJ*	YES	N	N	N	N	NO
464	N	*traT*	YES	N	N	N	N	NO
465	N	*traT*	YES	N	N	N	N	NO
475	N	N	NO	N	N	N	N	NO
477	N	*traT*	YES	N	N	N	N	NO
478	N	*traT*	YES	N	N	N	N	NO
481	N	*traT*	NO	N	N	N	N	NO
483	N	*traT*	NO	N	N	N	N	NO
484	N	*traT*	NO	N	N	N	N	NO
485	N	*traT*	YES	N	N	N	N	NO
486	N	*traT*	YES	N	N	N	N	NO
487	N	*traT*	NO	N	N	N	N	NO
489	N	*traT*	NO	N	N	N	N	NO
492	N	*traT*	NO	N	N	N	N	NO
494	N	*traT*	YES	N	N	N	N	NO
495	N	*traT*	NO	N	N	N	N	NO
498	*pilJPM*	*traTJ*	NO	*cia*	*cib*	N	N	NO
499	N	*traT*	NO	N	N	N	N	NO
500	N	*traT*	NO	N	N	N	N	NO
696	N	*traT*	NO	N	N	N	N	NO
710	N	*traT*	YES	N	N	N	N	NO
N028	N	*traTJ*	NO	*cia*	*cib*	*cvaAB*	*imm*	YES
N029	N	*traTJ*	YES	*cia*	*cib*	*cvaAB*	*imm*	YES
N030	*pilJPM*	*traTJ*	YES	*cia*	*cib*	*cvaAB*	*imm*	YES
N032	N	*traTJ*	NO	*cia*	*cib*	*cvaAB*	*imm*	YES
N033	N	*traTJ*	NO	*cia*	*cib*	*cvaAB*	*imm*	YES
N034	*pilJPM*	*traTJ*	YES	*cia*	*cib*	*cvaAB*	*imm*	YES
N035	N	*traTJ*	NO	*cia*	*cib*	*cvaAB*	*imm*	YES
N061	*pilJPM*	*traTJ*	NO	*cia*	*cib*	N	*imm*	NO
N062	*pilJPM*	*traTJ*	NO	*cia*	*cib*	N	*imm*	NO
N063	*pilJPM*	*traTJ*	NO	*cia*	*cib*	N	*imm*	NO
N065	*pilJPM*	*traTJ*	NO	*cia*	*cib*	N	*imm*	YES
N066	*pilJPM*	*traTJ*	NO	*cia*	*cib*	N	*imm*	NO
N067	*pilJPM*	*traTJ*	NO	*cia*	*cib*	N	*imm*	NO
N068	*pilJPM*	*traTJ*	NO	*cia*	*cib*	N	*imm*	YES
N069	*pilJPM*	*traTJ*	YES	*cia*	*cib*	*cvaABC*	*imm*	YES
N070	*pilJPM*	*traTJ*	NO	*cia*	*cib*	N	*imm*	NO
N071	*pilJPM*	*traTJ*	NO	*cia*	*cib*	N	*imm*	YES
N073	*pilJPM*	*traTJ*	NO	*cia*	*cib*	N	*imm*	YES
N075	*pilJPM*	*traTJ*	NO	*cia*	*cib*	N	*imm*	NO
N135	*pilJPM*	*traTJ*	NO	*cia*	*cib*	*cvaABC*	*imm*	YES
N138	*pilJPM*	*traTJ*	NO	*cia*	*cib*	*cvaABC*	*imm*	YES
N140	*pilJPM*	*traTJ*	NO	*cia*	*cib*	*cvaABC*	*imm*	YES

N: indicates absence of the gene.

**Table 3:** In silico genome analysis of isolates able to transfer plasmids and antimicrobial resistance genes to recipients and able to generate transconjugants.

Donor	PlasmidFinder	ResFinder (Resistance Genes)	Transconjugant	PlasmidFinder	ResFinder (Resistance Genes)
163	IncFIB, IncFIA, IncA/C2, IncFII(pCoo), IncX4, IncI1	*blaCMY-2, blaTEM-1B, sul1, sul2, aac(3)-VIa, aadA1, ant(2″)-Ia, aph(3″)-Ib, aph(3′)-Ia, aph(6)-Id, cmlA1, floR, tet(A), tet(B)*	X163	IncI1, IncX4	*sul1, aac(3)-VIa, aadA1*
375	IncFIB, ColpVC, IncA/C2, IncFII(S), IncI1	*sul1, sul2, aac(3)-VIa, aadA12, aph(3″)-Ib, aph(3′)-Ia, aph(6)-Id, tet(A), blaCMY-2, floR*	X375	IncA/C2	*sul2, blaCMY-2, tet(A), aph(3″)-Ib, aph(6)-Id*
393	IncFIB, ColpVC, IncFIA, IncFII, IncI1	*blaTEM-1B, blaTEM, aph(3′)-Ia, aadA12, tet(A), sul1*	X393	IncFIB, IncFIA, IncFII	*tet(A), sul1, aadA12, aph(3′)-Ia*
443	IncFIB, IncA/C2, IncFIA, IncFII(pCoo)	*ant(2″)-Ia, aph(3″)-Ib, aph(3′)-Ia, aph(6)-Id, blaCMY-2, blaTEM-1B, cmlA1, floR, tet(A), tet(B), sul2*	X443	IncFIB, IncFIA, IncFII(pcoo)	*aph(3″)-Ib, aph(3′)-Ia, aph(6)-Id, sul2, tet(B)*
447	IncFIB, ColpVC, IncA/C2, IncFIA, IncFII(pCoo)	*aph(3″)-Ib, aph(3′)-Ia, aph(6)-Id, blaCMY-2, floR, tet(A), tet(B), sul2*	X447	IncFIB, IncFIA, IncFII(pcoo)	*tet(B), sul2, aph(3″)-Ib, aph(3′)-Ia, aph(6)-Id*
452	IncFIB, ColpVC, IncFIA, IncFII(pCoo), IncA/C2	*blaCMY-2, blaTEM, tet(A), tet(B), sul2, ant(2″)-Ia, aph(3″)-Ib, aph(3″)-Ib, aph(3′)-Ia, aph(6)-Id, cmlA1, floR*	X452	IncFIB, IncFIA, IncFII(pcoo)	*aph(3″)-Ib, aph(3′)-Ia, aph(6)-Id, sul2, tet(B)*
457	IncFIB, ColpVC, IncFIA, IncFII(pCoo)	*aph(3″)-Ib, aph(3′)-Ia, aph(6)-Id, blaCMY-2, floR, tet(A), tet(B), sul2*	X457	IncFIB, IncFIA, IncFII(pcoo)	*sul2, aph(3″)-Ib, tet(B)*
458	IncFIB, ColpVC, IncFIA, IncFII, IncI1	*blaTEM-1B*	X458	IncFIB, IncFIA, IncFII	*blaTEM-1B*
459	IncFIB, Col156, IncFIC(FII), IncI1	*blaCMY-2*	X459	IncI1	*blaCMY-2*
460	IncFIB, ColpVC, IncA/C2, IncFIA, IncFII(pCoo)	*aph(3″)-Ib, aph(3′)-Ia, aph(6)-Id, blaCMY-2, floR, tet(A), tet(B), sul2*	X460	IncFIB, IncFIA, IncFII(pcoo)	*aph(3″)-Ib, aph(3′)-Ia, aph(6)-Id, sul2, tet(B)*
462	IncFIB, IncA/C2, IncFIA, IncFII(pCoo)	*blaCMY-2, blaTEM-1B, sul2, tet(A), tet(B), cmlA1, floR, ant(2″)-Ia, aph(3″)-Ib, aph(3′)-Ia, aph(6)-Id*	X462	IncFIB, IncFIA, IncFII(pcoo)	*sul2, aph(3″)-Ib, aph(3′)-Ia, aph(6)-Id, tet(B)*
463	IncFIB, ColpVC, IncFII, IncI1, Col156, IncFIA	*sul1, aadA12, aph(3′)-Ia, tet(A), blaTEM-1B*	X463	IncFIB, IncFIA, IncFII	*aadA12, aph(3′)-Ia, blaTEM-1B, sul1, tet(A)*
464	IncFIB, ColpVC, IncA/C2, IncFIA, IncFII(pCoo)	*ant(2″)-Ia, aph(3″)-Ib, aph(3′)-Ia, aph(6)-Id, blaCMY-2, blaTEM-1B, cmlA1, floR, tet(A), tet(B), sul2*	X464	IncFIB, IncFIA, IncFII(pcoo)	*aph(3″)-Ib, aph(3′)-Ia, aph(6)-Id, sul2, tet(B)*
465	IncFIB, IncA/C2, IncFIA, IncFII(pCoo)	*aac(6′)-Iaa, aph(3″)-Ib, aph(3′)-Ia, aph(6)-Id, blaCMY-2, floR, tet(A), tet(B), sul2*	X465	IncFIB, IncFIA, IncFII(pcoo)	*aph(3″)-Ib, aph(3′)-Ia, aph(6)-Id, sul2, tet(B)*
477	IncFIB, ColpVC, IncA/C2, IncFIA, IncFII(pCoo)	*aph(3″)-Ib, aph(3′)-Ia, aph(6)-Id, blaCMY-2, floR, tet(A), tet(B), sul2*	X477	IncFIB, IncFIA, IncFII(pcoo)	*aph(3″)-Ib, aph(3′)-Ia, aph(6)-Id, sul2*
478	IncFIB, IncA/C2, IncFIA, IncFII(pCoo)	*aadA1, aadA12, aadA21, aadA22, aph(3″)-Ib, aph(3′)-Ia, aph(6)-Id, blaCMY-2, blaTEM-1B, cmlA1, floR, tet(A), tet(B), sul2*	X478	IncFIB, IncFIA, IncFII(pcoo), IncA/C2	*cmlA1, floR, tet(A), tet(B), sul2, aph(3″)-Ib, aph(6)-Id, blaCMY-2, blaTEM-1B*
485	IncFIB, IncA/C2, IncFIA, IncFII(pCoo)	*ant(2″)-Ia, aph(3″)-Ib, aph(3′)-Ia, aph(6)-Id, blaCMY-2, blaTEM-1B, cmlA1, floR, tet(A), tet(B), sul2*	X485	IncFIB, IncFIA, IncFII(pcoo)	*tet(B), sul2, aph(3″)-Ib, aph(3′)-Ia, aph(6)-Id*
486	IncFIB, IncA/C2, IncFIA, IncFII(pCoo)	*ant(2″)-Ia, aph(3″)-Ib, aph(3′)-Ia, aph(6)-Id, blaCMY-2, blaTEM-1B, cmlA1, floR, tet(A), tet(B), sul2*	X486	IncFIB, IncFIA, IncFII(pcoo))	*aph(3″)-Ib, aph(3′)-Ia, aph(6)-Id, sul2, tet(B)*
494	IncFIB, IncFII(S), IncA/C2	*blaCMY-2, blaTEM-1B, sul2, sul1 tet(A), floR, ant(2″)-Ia, aph(3″)-Ib, aph(3′)-Ia, aph(6)-Id*	X494	IncA/C2	*blaCMY-2, tet(A), floR, aph(3″)-Ib, aph(6)-Id, sul2*
710	IncFIB, IncFIA, IncA/C2, IncFII(pCoo), IncX4	*ant(2″)-Ia, aph(3″)-Ib, aph(6)-Id, cmlA1, floR, tet(A), tet(B), sul2, blaCMY-2, blaTEM-1B*	X710	IncFIB, IncFIA, IncFII(pcoo)	*tet(B), aph(3″)-Ib, aph(3′)-Ia, aph(6)-Id, sul2*
N029	IncFIB, ColpVC, IncFIC(FII), IncHI2, IncHI2A	*aph(3″)-Ib, aph(6)-Id, tet(B), blaCMY-2*	XN029	IncFIB	*aph(3″)-Ib, aph(6)-Id, tet(B)*
N030	IncFIB, ColpVC, IncFIC(FII), IncI1	*aph(3″)-Ib, blaCMY-2*	XN030	ColpVC, IncI1	*aph(3″)-Ib, blaCMY-2*
N034	IncFIB, ColpVC, IncFIC(FII), IncI1	*aph(3″)-Ib, blaCMY-2*	XN034	ColpVC, IncI1	*aph(3″)-Ib, blaCMY-2*
N069	IncFIB, ColpVC, IncFIC(FII), IncI1	*aadA1, sul1*	XN069	IncI1	*aac(3)-VIa, aadA1, sul1*

**Table 4:** In silico genome analysis for isolates that were able to transfer virulence genes to recipients and generate transconjugants.

Donor	Iron Acquisition Genes				*Salmonella* Plasmid Virulence (*spv*)	Transconjugant	Iron Acquisition Genes				*Salmonella* Plasmid Virulence (*spv*)
	*iuc*	*iut*	*iro*	*sit*			*iuc*	*iut*	*iro*	*sit*	
163	*iucABCD*	*iutA*	*iroNB*	*sitA*	N	X163	N	N	N	N	N
375	N	*iutA*	*iroNB*	*sitA*	*spv*	X375	N	N	N	N	N
393	N	*iutA*	*iroNBC*	*sitA*	N	X393	N	*iutA*	N	N	N
443	*iucABCD*	*iutA*	*iroNB*	*sitAB*	N	X443	*iucABCD*	*iutA*	N	*sitABCD*	N
447	*iucABCD*	*iutA*	*iroNB*	*sitAB*	N	X447	*iucABCD*	*iutA*	N	*sitABCD*	N
452	*iucABCD*	*iutA*	*iroNB*	*sitAB*	N	X452	*iucABCD*	*iutA*	N	*sitABCD*	N
457	*iucABCD*	*iutA*	*iroNB*	*sitAB*	N	X457	*iucABCD*	*iutA*	N	*sitABCD*	N
458	N	*iutA*	*iroNB*	*sitA*	N	X458	N	*iutA*	N	N	N
459	*iucABCD*	*iutA*	*iroNBCD*	*sitAB*	N	X459	N	N	N	N	N
460	*iucABCD*	*iutA*	*iroNB*	*sitA*	N	X460	*iucABCD*	N	N	*sitABCD*	N
462	*iucABCD*	*iutA*	*iroNB*	*sitAB*	N	X462	*iucABCD*	*iutA*	N	*sitABCD*	N
463	N	*iutA*	*iroNB*	*sitA*	N	X463	N	*iutA*	N	N	N
464	*iucABCD*	*iutA*	*iroNB*	*sitAB*	N	X464	*iucABCD*	*iutA*	N	*sitABCD*	N
465	*iucABCD*	*iutA*	*iroNB*	*sitAB*	N	X465	*iucABCD*	*iutA*	N	*sitABCD*	N
477	*iucABCD*	*iutA*	*iroNB*	*sitAB*	N	X477	*iucABCD*	*iutA*	N	*sitABCD*	N
478	*iucABCD*	*iutA*	*iroNB*	*sitAB*	N	X478	*iucABCD*	*iutA*	N	*sitABCD*	N
485	*iucABCD*	*iutA*	*iroNB*	*sitAB*	N	X485	*iucABCD*	*iutA*	N	*sitABCD*	N
486	*iucABCD*	*iutA*	*iroNB*	*sitAB*	N	X486	*iucABCD*	*iutA*	N	*sitABCD*	N
494	N	N	*iroNB*	*sitA*	*spv*	X494	N	N	N	N	N
710	*iucABCD*	*iutA*	*iroNB*	*sitAB*	N	X710	*iucABCD*	*iutA*	N	*sitABCD*	N
N029	*iucABCD*	*iutA*	*iroNBCD*	*sitAB*	N	XN029	*iucACD*	*iutA*	N	N	N
N030	*iucABCD*	*iutA*	*iroNBCD*	*sitAB*	N	XN030	N	N	N	N	N
N034	*iucABCD*	*iutA*	*iroNBCD*	*sitAB*	N	XN034	N	N	N	N	N
N069	*iucABCD*	*iutA*	*iroNBCD*	*sitAB*	N	XN069	N	N	N	N	N

N: indicates absence of the gene.

## Data Availability

All sequences generated for this project can be found in GenBank, under the accession numbers listed in Table 1.

## Supplementary Materials

The following are available online at https://www.mdpi.com/2073-4425/11/11/1307/s1, Figure S1: SNP-based phylogenetic tree analysis for 79 *S*. Typhimurium. The isolate number/names are followed by the year of isolation, Table S1: Iron acquisition system genes and SPI-1 and SPI-2 encoded T3SS genes found in the 71 S. Typhimurium isolates using PATRIC, Table S2: Additional reference *S.* Typhimurium isolates including their plasmid types and antimicrobial resistance genes of the more recently sequenced, Table S3: Spreadsheet displaying the results of the NCTR Virulence Factor Database gene detection for the *S.* Typhimurium isolates included in the study.
