# Supplementary material for: Genotypic and Phenotypic Characterization of Incompatibility Group FIB Positive Salmonella enterica Serovar Typhimurium Isolates from Food Animal Sources
Source: Genes (Basel). 2020 Nov 4;11(11):1307. doi: 10.3390/genes11111307 (PMC7716204; doi:10.3390/genes11111307)
Supplement: Supplementary file 1 [file genes-11-01307-s001.zip › genes-950884-supplementary/genes-950884 suplymentary.docx]

**Table S1:** Iron acquisition system genes and SPI-1 and SPI-2 encoded T3SS genes found in the 71 *S.* Typhimurium isolates using PATRIC

| **Isolates** | ***Salmonella* Plasmid Virulence** | **Iron acquisition and Regulator System Genes** | | | | ***S*PI-1 T3SS** | | ***S*PI-2 T3SS** | | | | | |
| --- | --- | --- | --- | --- | --- | --- | --- | --- | --- | --- | --- | --- | --- |
| 163 | N | *iucABCD* | *iutA* | *iroNB* | *sitABCD* | *sipABCDF* | *sopADE2* | *spiCR* | *sseFGIJBDCE* | *sifA* | *sspA* | *pipABCD* | *sopD2* |
| 368 | *spv* | N | *iutA* | *iroNB* | *sitAB* | *sipABCDF* | *sopADE2* | *spiCR* | *sseFGIJBDCE* | *sifA* | *sspA* | *pipABCD* | *sopD2* |
| 373 | *spv* | N | *iutA* | *iroNB* | *sitAB* | *sipABCDF* | *sopADE2* | *spiCR* | *sseFGIJBDCE* | *sifA* | *sspA* | *pipABCD* | *sopD2* |
| 374 | *spv* | N | *iutA* | *iroNB* | *sitAB* | *sipABCDF* | *sopADE2* | *spiCR* | *sseFGIJBDCE* | *sifA* | *sspA* | *pipABCD* | *sopD2* |
| 375 | *spv* | N | *iutA* | *iroNB* | *sitAB* | *sipABCDF* | *sopADE2* | *spiCR* | *sseFGIJBDCE* | *sifA* | *sspA* | *pipABCD* | *sopD2* |
| 376 | N | *iucABCD* | *iutA* | *iroNB* | *sitABCD* | *sipABCDF* | *sopADE2* | *spiCR* | *sseFGIJBDCE* | *sifA* | *sspA* | *pipABCD* | *sopD2* |
| 391 | N | *iucABCD* | *iutA* | *iroNBCD* | *sitABCD* | *sipABCDF* | *sopADE2* | *spiCR* | *sseFGIJBDCE* | *sifA* | *sspA* | *pipABCD* | *sopD2* |
| 393 | N | N | *iutA* | *iroNBC* | *sitAB* | *sipABCDF* | *sopADE2* | *spiCR* | *sseFGJBDCE* | *sifA* | *sspA* | *pipABCD* | *sopD2* |
| 397 | N | *iucABCD* | *iutA* | *iroNB* | *sitABCD* | *sipABCDF* | *sopADE2* | *spiCR* | *sseFGIJBDCE* | *sifA* | *sspA* | *pipABCD* | *sopD2* |
| 411 | N | *iucABCD* | *iutA* | *iroNBC* | *sitABCD* | *sipABCDF* | *sopADE2* | *spiCR* | *sseFGIJBDCE* | *sifA* | *sspA* | *pipABCD* | *sopD2* |
| 417 | N | *iucABCD* | *iutABCD* | *iroNB* | *sitABCD* | *sipABCDF* | *sopADE2* | *spiCR* | *sseFGIJBDCE* | *sifA* | *sspA* | *pipABCD* | *sopD2* |
| 419 | N | N | N | *iroNB* | *sitABCD* | *sipABCDF* | *sopADE2* | *spiCR* | *sseFGIJBDCE* | *sifA* | *sspA* | *pipABCD* | *sopD2* |
| 420 | N | *iucABCD* | *iutA* | *iroNBCD* | *sitABCD* | *sipABCDF* | *sopADE2* | *spiCR* | *sseFGIJBDCE* | *sifA* | *sspA* | *pipABCD* | *sopD2* |
| 421 | N | *iucABCD* | *iutA* | *iroNBCD* | *sitABCD* | *sipABCDF* | *sopADE2* | *spiCR* | *sseFGIJBDCE* | *sifA* | *sspA* | *pipABCD* | *sopD2* |
| 423 | N | *iucABCD* | *iutA* | *iroNBCD* | *sitABCD* | *sipABCDF* | *sopADE2* | *spiCR* | *sseFGIJBDCE* | *sifA* | *sspA* | *pipABCD* | *sopD2* |
| 426 | N | *iucABCD* | *iutA* | *iroNBCD* | *sitABCD* | *sipABCDF* | *sopADE2* | *spiCR* | *sseFGIJBDCE* | *sifA* | *sspA* | *pipABCD* | *sopD2* |
| 427 | N | *iucABCD* | *iutA* | *iroNBCD* | *sitABCD* | *sipABCDF* | *sopADE2* | *spiCR* | *sseFGIJBDCE* | *sifA* | *sspA* | *pipABCD* | *sopD2* |
| 443 | N | *iucABCD* | *iutA* | *iroNB* | *sitABCD* | *sipABCDF* | *sopADE2* | *spiCR* | *sseFGIJBDCE* | *sifA* | *sspA* | *pipABCD* | *sopD2* |
| 447 | N | *iucABCD* | *iutA* | *iroNB* | *sitABCD* | *sipABCDF* | *sopADE2* | *spiCR* | *sseFGIJBDCE* | *sifA* | *sspA* | *pipABCD* | *sopD2* |
| 448 | N | *iucABCD* | *iutA* | *iroNBCD* | *sitABCD* | *sipABCDF* | *sopADE2* | *spiCR* | *sseFGIJBDCE* | *sifA* | *sspA* | *pipABCD* | *sopD2* |
| 449 | N | *iucABCD* | *iutA* | *iroNBCD* | *sitABCD* | *sipABCDF* | *sopADE2* | *spiCR* | *sseFGIJBDCE* | *sifA* | *sspA* | *pipABCD* | *sopD2* |
| 452 | N | *iucABCD* | *iutA* | *iroNB* | *sitABCD* | *sipABCDF* | *sopADE2* | *spiCR* | *sseFGIJBDCE* | *sifA* | *sspA* | *pipABCD* | *sopD2* |
| 455 | *spv* | N | *iutA* | *iroNB* | *sitAB* | *sipABCDF* | *sopADE2* | *spiCR* | *sseFGIJBDCE* | *sifA* | *sspA* | *pipABCD* | *sopD2* |
| 457 | N | *iucABCD* | *iutA* | *iroNB* | *sitABCD* | *sipABCDF* | *sopADE2* | *spiCR* | *sseFGIJBDCE* | *sifA* | *sspA* | *pipABCD* | *sopD2* |
| 458 | N | N | *iutA* | *iroNB* | *sitAB* | *sipABCDF* | *sopADE2* | *spiCR* | *sseFGJBDCE* | *sifA* | *sspA* | *pipABCD* | *sopD2* |
| 459 | N | *iucABCD* | *iutA* | *iroNBCD* | *sitABCD* | *sipABCDF* | *sopADE2* | *spiCR* | *sseFGIJBDCE* | *sifA* | *sspA* | *pipABCD* | *sopD2* |
| 460 | N | *iucABCD* | *iutA* | *iroNB* | *sitABCD* | *sipABCDF* | *sopADE2* | *spiCR* | *sseFGIJBDCE* | *sifA* | *sspA* | *pipABCD* | *sopD2* |
| 462 | N | *iucABCD* | *iutA* | *iroNB* | *sitABCD* | *sipABCDF* | *sopADE3* | *spiCR* | *sseFGIJBDCE* | *sifA* | *sspA* | *pipABCD* | *sopD2* |
| 463 | N | N | *iutA* | *iroNB* | *sitAB* | *sipABCDF* | *sopADE2* | *spiCR* | *sseFGIJBDCE* | *sifA* | *sspA* | *pipABCD* | *sopD2* |
| 464 | N | *iucABCD* | *iutA* | *iroNB* | *sitABCD* | *sipABCDF* | *sopADE2* | *spiCR* | *sseFGIJBDCE* | *sifA* | *sspA* | *pipABCD* | *sopD2* |
| 465 | N | *iucABCD* | *iutA* | *iroNB* | *sitABCD* | *sipABCDF* | *sopADE2* | *spiCR* | *sseFGIJBDCE* | *sifA* | *sspA* | *pipABCD* | *sopD2* |
| 475 | *spv* | N | N | *iroNB* | *sitAB* | *sipABCDF* | *sopADE2* | *spiC* | *sseFGIJBDCE* | *sifA* | *sspA* | *pipABCD* | *sopD2* |
| 477 | N | *iucABCD* | *iutA* | *iroNB* | *sitABCD* | *sipABCDF* | *sopADE2* | *spiCR* | *sseFGIJBDCE* | *sifA* | *sspA* | *pipABCD* | *sopD2* |
| 478 | N | *iucABCD* | *iutA* | *iroNB* | *sitABCD* | *sipABCDF* | *sopADE2* | *spiCR* | *sseFGIJBDCE* | *sifA* | *sspA* | *pipABCD* | *sopD2* |
| 481 | N | *iucABCD* | *iutA* | *iroNB* | *sitABCD* | *sipABCDF* | *sopADE2* | *spiCR* | *sseFGIJBDCE* | *sifA* | *sspA* | *pipABCD* | *sopD2* |
| 483 | N | *iucABCD* | *iutA* | *iroNB* | *sitABCD* | *sipABCDF* | *sopADE2* | *spiCR* | *sseFGJBDCE* | *sifA* | *sspA* | *pipABCD* | *sopD2* |
| 484 | N | *iucABCD* | *iutA* | *iroNB* | *sitABCD* | *sipABCDF* | *sopADE2* | *spiCR* | *sseFGIJBDCE* | *sifA* | *sspA* | *pipABCD* | *sopD2* |
| 485 | N | *iucABCD* | *iutA* | *iroNB* | *sitABCD* | *sipABCDF* | *sopADE2* | *spiCR* | *sseFGIJBDCE* | *sifA* | *sspA* | *pipABCD* | *sopD2* |
| 486 | N | *iucABCD* | *iutA* | *iroNB* | *sitABCD* | *sipABCDF* | *sopADE2* | *spiCR* | *sseFGIJBDCE* | *sifA* | *sspA* | *pipABCD* | *sopD2* |
| 487 | *spv* | N | *iutA* | *iroNB* | *sitAB* | *sipABCDF* | *sopADE2* | *spiCR* | *sseFGIJBDCE* | *sifA* | *sspA* | *pipABCD* | *sopD2* |
| 489 | *spv* | N | *iutA* | *iroNB* | *sitAB* | *sipABCDF* | *sopADE2* | *spiCR* | *sseFGIJBDCE* | *sifA* | *sspA* | *pipABCD* | *sopD2* |
| 492 | *spv* | N | *iutA* | *iroNB* | *sitAB* | *sipABCDF* | *sopADE2* | *spiCR* | *sseFGIJBDCE* | *sifA* | *sspA* | *pipABCD* | *sopD2* |
| 494 | *spv* | N | N | *iroNB* | *sitAB* | *sipABCDF* | *sopADE2* | *spiCR* | *sseFGIJBDCE* | *sifA* | *sspA* | *pipABCD* | *sopD2* |
| 495 | *spv* | N | *iutA* | *iroNB* | *sitAB* | *sipABCDF* | *sopADE2* | *spiCR* | *sseFGIJBDCE* | *sifA* | *sspA* | *pipABCD* | *sopD2* |
| 498 | *spv* | N | *iutA* | *iroNB* | *sitAB* | *sipABCDF* | *sopADE2* | *spiCR* | *sseFGIJBDCE* | *sifA* | *sspA* | *pipABCD* | *sopD2* |
| 499 | *spv* | N | *iutA* | *iroNB* | *sitAB* | *sipABCDF* | *sopADE2* | *spiCR* | *sseFGIJBDCE* | *sifA* | *sspA* | *pipABCD* | *sopD2* |
| 500 | *spv* | N | *iutA* | *iroNB* | *sitAB* | *sipABCDF* | *sopADE2* | *spiCR* | *sseFGIJBDCE* | *sifA* | *sspA* | *pipABCD* | *sopD2* |
| 696 | N | *iucABCD* | *iutA* | *iroNB* | *sitABCD* | *sipABCDF* | *sopADE2* | *spiCR* | *sseFGIJBDCE* | *sifA* | *sspA* | *pipABCD* | *sopAE2D2* |
| 710 | N | *iucABCD* | *iutA* | *iroNB* | *sitABCD* | *sipABCDF* | *sopADE3* | *spiCR* | *sseFGIJBDCE* | *sifA* | *sspA* | *pipABCD* | *sopAE2D2* |
| N028 | N | *iucABCD* | *iutA* | *iroNBCD* | *sitABCD* | *sipABCDF* | *sopADE2* | *spiCR* | *sseFGIJBDCE* | *sifA* | *sspA* | *pipABCD* | *sopD2* |
| N029 | N | *iucABCD* | *iutA* | *iroNBCD* | *sitABCD* | *sipABCDF* | *sopADE2* | *spiCR* | *sseFGIJBDCE* | *sifA* | *sspA* | *pipABCD* | *sopD2* |
| N030 | N | *iucABCD* | *iutA* | *iroNBCD* | *sitABCD* | *sipABCDF* | *sopADE2* | *spiCR* | *sseFGIJBDCE* | *sifA* | *sspA* | *pipABCD* | *sopD2* |
| N032 | N | *iucABCD* | *iutA* | *iroNBCD* | *sitABCD* | *sipABCDF* | *sopADE2* | *spiCR* | *sseFGIJBDCE* | *sifA* | *sspA* | *pipABCD* | *sopD2* |
| N033 | N | *iucABCD* | *iutA* | *iroNBCD* | *sitABCD* | *sipABCDF* | *sopADE2* | *spiCR* | *sseFGIJBDCE* | *sifA* | *sspA* | *pipABCD* | *sopD2* |
| N034 | N | *iucABCD* | *iutA* | *iroNBCD* | *sitABCD* | *sipABCDF* | *sopADE2* | *spiCR* | *sseFGIJBDCE* | *sifA* | *sspA* | *pipABCD* | *sopD2* |
| N035 | N | *iucABCD* | *iutA* | *iroNBCD* | *sitABCD* | *sipABCDF* | *sopADE2* | *spiCR* | *sseFGIJBDCE* | *sifA* | *sspA* | *pipABCD* | *sopD2* |
| N061 | N | *iucABCD* | *iutA* | *iroNB* | *sitABCD* | *sipABCDF* | *sopADE2* | *spiCR* | *sseFGIJBDCE* | *sifA* | *sspA* | *pipABCD* | *sopD2* |
| N062 | N | *iucABCD* | *iutA* | *iroNB* | *sitABCD* | *sipABCDF* | *sopADE2* | *spiCR* | *sseFGIJBDCE* | *sifA* | *sspA* | *pipABCD* | *sopD2* |
| N063 | N | *iucABCD* | *iutA* | *iroNB* | *sitABCD* | *sipABCDF* | *sopADE2* | *spiCR* | *sseFGIJBDCE* | *sifA* | *sspA* | *pipABCD* | *sopD2* |
| N065 | N | *iucABCD* | *iutA* | *iroNB* | *sitABCD* | *sipABCDF* | *sopADE2* | *spiCR* | *sseFGIJBDCE* | *sifA* | *sspA* | *pipABCD* | *sopD2* |
| N066 | N | *iucABCD* | *iutA* | *iroNB* | *sitABCD* | *sipABCDF* | *sopADE2* | *spiCR* | *sseFGIJBDCE* | *sifA* | *sspA* | *pipABCD* | *sopD2* |
| N067 | N | *iucABCD* | *iutA* | *iroNB* | *sitABCD* | *sipABCDF* | *sopADE2* | *spiCR* | *sseFGIJBDCE* | *sifA* | *sspA* | *pipABCD* | *sopD2* |
| N068 | N | *iucABCD* | *iutA* | *iroNB* | *sitABCD* | *sipABCDF* | *sopADE2* | *spiCR* | *sseFGIJBDCE* | *sifA* | *sspA* | *pipABCD* | *sopD2* |
| N069 | N | *iucABCD* | *iutA* | *iroNBCD* | *sitABCD* | *sipABCDF* | *sopADE2* | *spiCR* | *sseFGIJBDCE* | *sifA* | *sspA* | *pipABCD* | *sopD2* |
| N070 | N | *iucABCD* | *iutA* | *iroNB* | *sitABCD* | *sipABCDF* | *sopADE2* | *spiCR* | *sseFGIJBDCE* | *sifA* | *sspA* | *pipABCD* | *sopD2* |
| N071 | N | *iucABCD* | *iutA* | *iroNB* | *sitABCD* | *sipABCDF* | *sopADE2* | *spiCR* | *sseFGIJBDCE* | *sifA* | *sspA* | *pipABCD* | *sopD2* |
| N073 | N | *iucABCD* | *iutA* | *iroNB* | *sitABCD* | *sipABCDF* | *sopADE2* | *spiCR* | *sseFGIJBDCE* | *sifA* | *sspA* | *pipABCD* | *sopD2* |
| N075 | N | *iucABCD* | *iutA* | *iroNB* | *sitABCD* | *sipABCDF* | *sopADE2* | *spiCR* | *sseFGIJBDCE* | *sifA* | *sspA* | *pipABCD* | *sopD2* |
| N135 | N | *iucABCD* | *iutA* | *iroNBCD* | *sitABCD* | *sipABCDF* | *sopADE2* | *spiCR* | *sseFGIJBDCE* | *sifA* | *sspA* | *pipABCD* | *sopD2* |
| N138 | N | *iucABCD* | *iutA* | *iroNBCD* | *sitABCD* | *sipABCDF* | *sopADE2* | *spiCR* | *sseFGIJBDCE* | *sifA* | *sspA* | *pipABCD* | *sopD2* |
| N140 | N | *iucABCD* | *iutA* | *iroNBCD* | *sitABCD* | *sipABCDF* | *sopADE2* | *spiCR* | *sseFGIJBDCE* | *sifA* | *sspA* | *pipABCD* | *sopD2* |

N=Not detected.

**Table S2:** Additional reference *S.* Typhimurium isolates including their plasmid types and antimicrobial resistance genes of the more recently sequnced

| **GenBank accession number** | **Year Collected** | **Sources** | **Country** | **PlasmidFinder** | **Antimicrobial Resistance Genes (ResFinder)** |
| --- | --- | --- | --- | --- | --- |
| MKLU01000008.1 | 2005 | Chicken | USA | Col(pHAD28), IncC | *tet(B)* |
| MOCT01000007.1 | 2010 | Bovine | USA | ColpVC, IncFIB(S), IncFII(S) | NO |
| MXFR00000000 | 2011 | Chicken | USA | IncC, IncI1-I(Gamma) | *aph(3'')-Ib, aph(6)-Id, sul2, tet(A)* |
| NZ_JYZR00000000 | 2012 | Turkey | USA | IncI1-I(Gamma) | *aph(3'')-Ib, aph(6)-Id, blaTEM-1B, sul2, tet(B)* |
| AAKNWK000000000.1 | 2016 | Turkey | USA | ColpVC, IncC, IncHI2, IncHI2A | *aph(3'')-Ib, aph(6)-Id, blaCMY-2, sul2, tet(A), tet(B)* |
| AALTXO000000000.1 | 2016 | Swine | USA | IncFIB(S), IncFII(S) | *aadA2b, blaCARB-2, floR, sul1, tet(G)* |
| AAKMFC000000000 | 2018 | Turkey | USA | NO | NO |
| AAKRYX010000049.1 | 2018 | Swine | USA | IncFIB | *tet(B)* |

**Table S3:** Spreadsheet displaying the results of the NCTR Virulence Factor Database gene detection for the *S.* Typhimurium isolates included in the study. A red cell (1) indicated presence of the gene and a white cell (0) indicated that the gene was not detected. This data was used to generate the dengrogram in Figure 3. (Excel spreadsheet uploaded as a supplemental file)


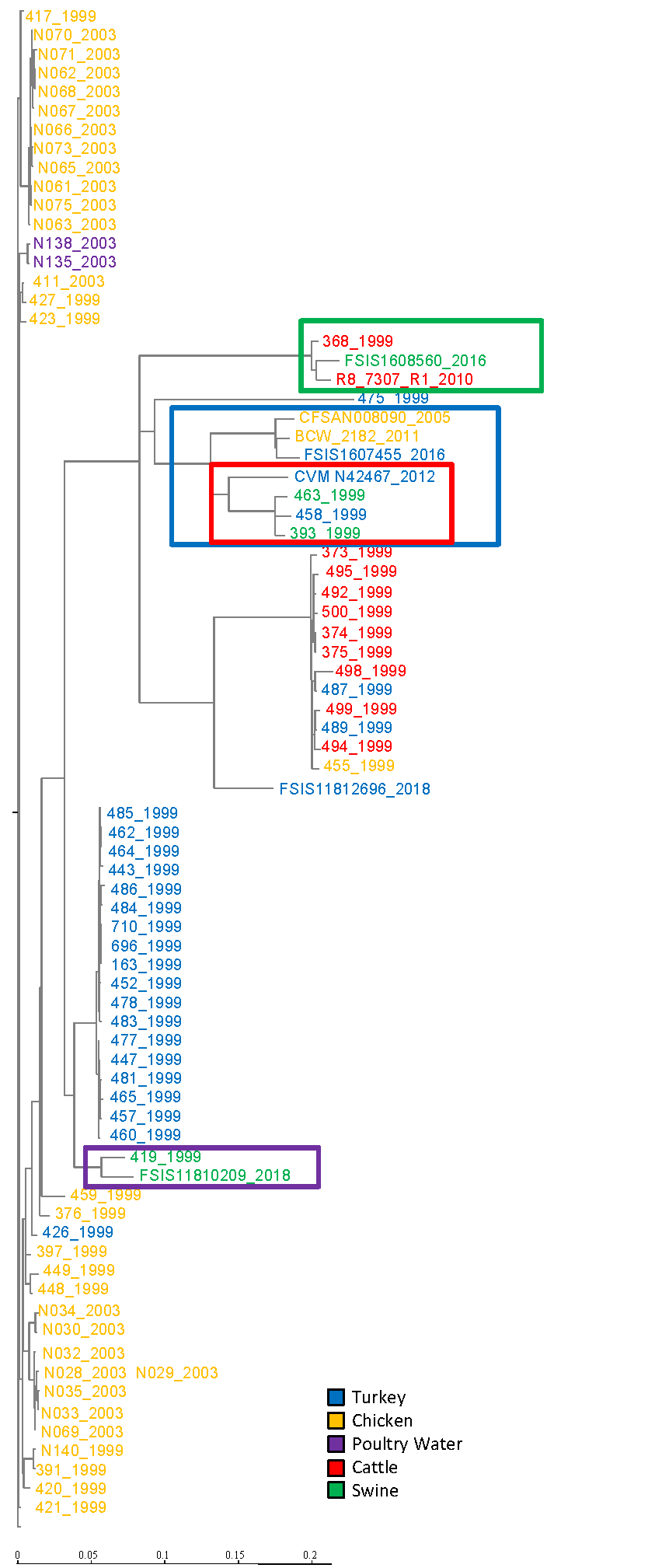


**Figure S1**: SNP-based phylogenetic tree analysis for 79 *S*. Typhimurium. The isolate number/names are followed by the year of isolation. The color boxes indicate the recent sequenced *S*. Typhimurium shared a high degree of genetic relatedness with this study and are described in the body of the manuscript. The numbers on the scale present the percentage of genetic variation.

.
